# Structural design of tetravalent T-cell engaging bispecific antibodies: improve developability by engineering disulfide bonds

**DOI:** 10.1186/s13036-021-00272-7

**Published:** 2021-06-29

**Authors:** Lin Yu, Nan Huang, Liangpeng Ge, Heng Sun, Yuna Fu, Chundong Liu, Jianhua Wang

**Affiliations:** 1 Key Laboratory of Biorheological Science and Technology (Ministry of Education), Chongqing University, No. 174 Shazheng Street, Shapingba District, 400044 Chongqing, China; 2 College of Bioengineering, Chongqing University, 400044 Chongqing, China; 3 Chongqing Academy of Animal Sciences, 402460 Chongqing, China; 4 Chongqing Engineering Technology Research Center for Medical Animal Resources Development and Application, 402460 Chongqing, China; 5 Qiuzhen College, Huzhou University, No.1 Xueshi Road, Wuxing District, 313000 Huzhou, China

**Keywords:** Bispecific antibody, ScFv, Disulfide bond, Stability

## Abstract

Since the advances in protein engineering and manufacture, over the last 30 years, antibody-based immunotherapeutic has become a powerful strategy to treat diseases. The T-cell engaging bispecific antibody (BsAb) by combining the Fab binding domain of tumor antigens and Fab or single-chain variable fragments (scFvs) binding domain of CD3 molecules, could redirect cytotoxic T cells to kill tumor cells. The IgG-scFv format of BsAb is a dual bivalent and asymmetrical design, which adds the benefit of potent cytotoxicity and less complicated for manufacture but limits the stability and production. Here, we engineered a series of interchain disulfide bonds in the Fab region of IgG-svFv BsAbs and evaluated its biophysical and biological properties. We found that simultaneously replaced the position of VH_44_-VL_100_ and CH1_126_-CL_121_ residues with cysteine, to form two additional disulfide bonds, could markedly increase monomeric BsAb formation and yield. The thermostability and stability against aggregation and degradation also performed better than BsAbs without extra disulfide bonds introduction. Besides, the affinity of engineered BsAbs was maintained, and the h8B-BsAb antibody had a slight enhancement in an inhibitory effect on target cells.

**Supplementary Information:**

The online version contains supplementary material available at 10.1186/s13036-021-00272-7.

## Introduction

Advance in biotechnology, the bispecific antibody (BsAb) that can bind two distinct epitopes, has become one of the most attractive therapeutic strategies in immunotherapy of cancer and other diseases. At present, two BsAbs (Blinatumomab and Emicizumab) are on the market, and more than 50 BsAbs are evaluating in clinic [1–3]. According to the action mechanism, the majority of BsAbs are designed to recruit immune cells (e.g., T cells), and part of BsAbs aim to block signaling pathways or delivery isotopes and drugs [3]. In particular, by combining tumor antigen binding specificity and T cell binding specificity into one molecule, 23 T-cell engaging bispecific antibodies are assessed in the clinical stage [4].

About a hundred different formats of BsAbs are reported and can roughly be classified into two categories based on lacking or possessing immunoglobulin G (IgG) Fc region [5]. BsAbs with IgG-Fc domains that can be recognized by neonatal receptor (FcRn) generally are stable and equipped with a long biological half-life in the serum [5]. Most tetravalent or multivalent bispecific antibodies are IgG like and symmetrical architecture. The antigen binding fragments (Fab) or single-chain variable fragment (scFv) connected to C-terminus/N-terminus of the heavy chain, the hinge region or light chain [6–11]. The IgG-scFv bispecific formats showed superior binding arm affinity or tumor cell lysis activity than other common tetravalent designs [8, 12, 13]. A recent study investigated antitumor activities when the anti-CD3 scFv domain fused to the C-terminus of light chains and heavy chains respectively, as well as under the different valency, which the light chain fused a scFv of tetravalent BsAb (IgG-like) had the best cytotoxicity against tumors [14].

However, the IgG-scFv format which the light chain extended with a scFv or a short peptide suffer from poor stability of bispecific antibody [8, 15], and our early experimental results also found that this format has been difficult to correctly assemble to expected monomers in the expression system, which the IgG geometric changes may affect the intrinsic disulfide bonds formation between heavy chain and light chain. Engineering the disulfide bonds are commonly utilized to enhancing the stability of protein including antibodies [16–19]. Introducing extra disulfide bond between the heavy (VH) and the light (VL)chain variable domains to stabilize the scFv binding domain [12, 20, 21], and even enhance the binding and antitumor activity [22].

Herein, we constructed a series of tetravalent IgG-scFv (light chain) antibodies introducing one or two additional disulfide bonds into the VH/VL domain or CH1/CL domain toward to CD3 and an alternative therapeutic target. We demonstrated that engineered disulfide bonds in the two domains could improve stabilities and amount of monomer, and the biological activities of these BsAbs maintained or enhanced.

## Materials and methods

### Cell culture

The HEK-293 F suspension cells (OPM Biosciences, China) were cultured in serum-free medium (OPM Biosciences) with 7 % CO2. The SK-GPC3 cells were constructed by our laboratory and cultured in DMEM complete growth medium with 10 % fetal bovine serum and1 % penicillin/streptomycin (Thermo Fisher Scientific, USA), which inserted human Glypican-3 genes into SK-Hep-1 cancer cells (National Infrastructure of Cell Line Resource, China). Human peripheral blood lymphocyte (PBMCs) were isolated from the blood of healthy donors by Ficoll® Paque Plus (GE Healthcare, USA) and cultured in RMPI-1640 complete growth medium (Thermo Fisher Scientific). Human blood samples were donated by healthy adult volunteers after informed consent. The study was approved for the collection of bloods by the ethics committee of The Army Medical University. All cells were incubated in a humidified atmosphere at 37 °C.

### Tetravalent BsAb design, expression and purification

All the tetravalent BsAbs consisted of heavy chains and light chains with coding sequences chemically synthesized (GENEWIZ, China) into pCDNA3.4 vector for transient expression by HEK-293 F cells. Specifically, the BsAbs were designed based on human IgG1, which the C-terminal of CL domain connected a CD3 binding domain (scFv) [23]using a (G_4_S)_3_ flexible linker. To improve the properly paired monomers, engineered disulfide bridges were added in Fab domains. Amino acid mutants of CH1 and CL regions need to meet the criteria of disulfide bonds formation with the distance of two α carbon atoms of cysteines (C_α_-C_α_) less than or equal to 7.0 Å and the two β carbon atoms of cysteines (C_β_- C_β_) less than or equal to 4.7 Å [24–26]. On the basis of crystal structure (6ATT.PDB) [27], seven paired mutants (A-G) of CH1 and CL interface were selected for experimental characterization: F126C/S121C, F126C/Q124C, P127C/S121C, A141C/F116C, H168C/T164C, F170C/T164C, V173C/Q160C (CH1/CL, Kabat numbering). Another engineered interchain disulfide bond (VH_44_-VL_100_) [20] was introduced in the VH/VL domain of h8B-BsAb-4, h8B-BsAb, CEA-BsAb and h2E-BsAb. The Fc region of BsAbs was designed to reduce the antibody-dependent cellular cytotoxicity and complement-dependent cytotoxicity [28, 29].

Expression titers of antibodies were measured by semiquantitative western blot [30] which was also used to analyze the monomers, dimers, polymers and unpaired chains of culture supernatants. Briefly, the cell cultural supernatants (day 7) were centrifuged and mixed with non-reducing LDS sample buffer (Thermo Fisher Scientific). A tetravalent purified BsAb (>97 % purity) with a series of concentration(6, 5, 4, 3, 2, 1, 0 ug) was used as standard antibodies, and was treated same with the cell supernatants. The mixtures were added into 4–12 % NuPAGE™ Bis-Tris Gels (Thermo Fisher Scientific) for sodium dodecyl sulfate polyacrylamide gel electrophoresis (SDS-PAGE). Proteins in gels then were transferred to polyvinylidene fluoride membrane by electrophoresis apparatus. After blocking by 5 % skim milk, the membrane was incubated with goat anti-human IgG (H + L) antibody- Alexa Fluor 633 (ratio of 1:5,000, Thermo Fisher Scientific) for 1 h at room temperature. Subsequently, the results were analyzed by ODYSSEY CLx and Image Studio (LI-COR, USA). Standard curve was drawn using gray values of protein bands and then was used to calculate the ratios of monomers and expression levels of BsAbs. As cell viability dropped to 60 %, cell supernatants were collected and filtered by 0.45 μm filter for purification using protein A affinity chromatography (GE Healthcare). Antibodies were analyzed by SDS-PAGE and stored in PBS buffer (pH 7.4) at -80℃.

### Thermal stability assay

Thermal Stability Measurement by differential scanning calorimetry (DSC) using a Microcal VP-DSC scanning microcalorimeter (MicroCal, UK). Briefly, BsAbs with 0.25 mg/ml were filtered by 0.22 μm filter and added to the sample wells. DSC measurements were performed at a 60 °C /h scan rate from 25 to 95 °C and data were analyzed by MicrolCal Origin 7.0 (Origin-Lab Corp.,MA). The T_m_ value indicated the midpoint temperature of the thermal unfolding transition of antibody.

### Shaking stability analysis

To test the ability of BsAbs to withstand mechanical stresses (e.g., shaking) during manufacturing and shipping, we performed turbidity assay and SDS-PAGE to evaluate the aggregation and degradation propensities [21]. The purified antibodies with 1 mg/mL were added to 2mL EP tubes and vortexed at 1400 rpm, 25ºC in a Thermomixer Comfort (Eppendorf, Germany) for up to 168 h. Antibody solutions were directly taken to measure the turbidity by recording the 595nm absorbance at 0, 2, 4, 24, 48, 96, 120, 168 h using a visible-ultraviolet spectrophotometer (BioTek, USA). The antibodies were also analyzed by SDS-PAGE after shaking 0 and 168 h.

### Antibody affinity measurement

The binding affinity of antibodies to antigens were measured by flow cytometry (FCM) and biolayer interferometry (BLI). For FCM, targeted cells (SK-GPC3, PBMCs) were washed and incubated with a dilution series of BsAbs for 1 h at 4ºC. The goat anti-human IgG(H + L)-Alexa Fluor 633 antibody (Thermo Fisher Scientific) was used as a secondary antibody to incubated with cells for 30 min at 4ºC. Washed cells were analyzed by the BD FACSVerse flow cytometer and BD FACSuite software (BD Bioscience, USA). Data of binding EC_50_ values were calculated by Prism Version 8.0 (GraphPad Software, USA). For BLI, antibodies with 20ug/mL were immobilized on anti-Human IgG Fc Capture (AHC) biosensors (Pall ForteBio, USA) for 60 s. Then the biosensors interacted with a series of concentrations antibodies (500nM, 250nM, 125nM, 62.5nM and 31.3nM) for 250 s and dissolved in PBS buffer (pH 7.4) for 250 s. Data acquisition and processing were conducted by an Octet Qke instrument and Data Analysis 7.0 (Pall ForteBio), respectively.

### Inhibition of cancer cells proliferation assay

Freshly isolated human PBMCs (effector cells, E) were mixed with SK-GPC3 cancer cells or SK-Hep-1 cells (target cells, T) by an E:T ratio of 10:1 and transferred to 96-plates for 2 h. Then a series of diluted antibodies were added in wells and incubation for 48 h at 37 ºC. Cell growth inhibition was measured by CCK-8 kit (Boster, China) according to the manufacturer’ s instructions. Data analysis performed using Prism Version 8.0, and the maximum efficiency was defined as the upper asymptote [31]. The SK-GPC3 cells are glypican-3 positive cells and SK-Hep-1 are Glypican-3 negative cells.

## Results

### Design of interchain disulfide bonds in CH1/CL domain of tetravalent T-cell engaging BsAbs

**Fig. 1:**
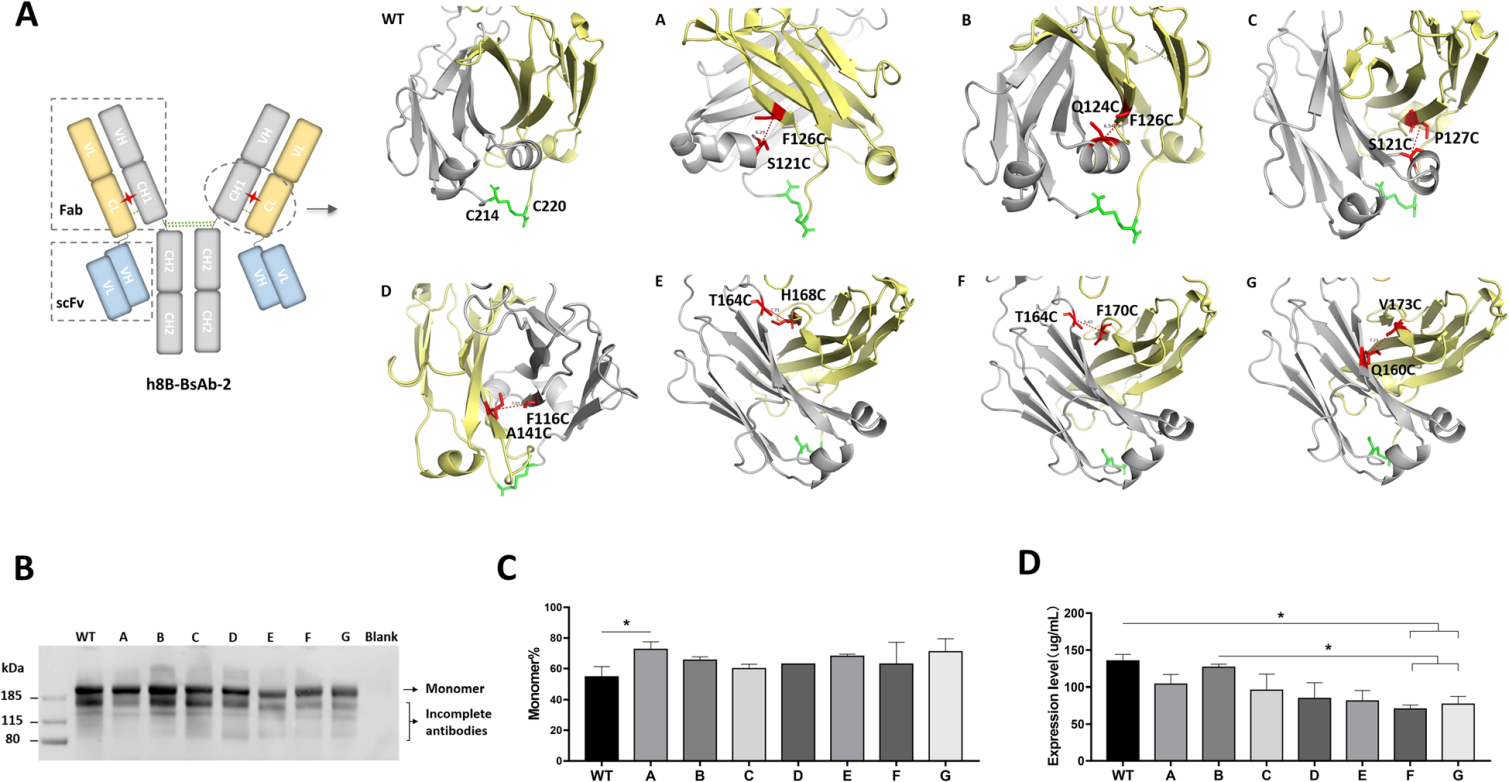
Design of single additional disulfide bond in CH1/CL domain of tetravalent BsAbs against glypican-3 and CD3 and its expression. (**A**) The schematic construction of tetravalent BsAb and crystal structure of CH1 and CL domain that are colored by yellow and gray. green: native disulfide bridges; red: engineered disulfide bridges. (**B**) Western blot (WB) of BsAbs expression. Blank: cell supernatant of a blank vector. Determination of monomeric BsAb (**C**) and expression levels (**D**). Mean ± SD (*n* = 3), *P < 0.05

The light chain of a fully human anti-glypican-3 antibody (IgG1, kappa) derived from our laboratory was connected to an anti-CD3 scFv binding domain [23] by a glycine-rich linker to form the tetravalent IgG-scFv bispecific antibody (theoretical molecular weight ~ 200 kDa). By retaining native interchain disulfide bridges between the heavy chain and light chain of IgG1, seven pairs of additional disulfide bonds (A-G) were introduced in CH1/CL constant domain, named h8B-BsAb-2 (Fig. 1 A). Amount of monomer of all BsAbs with engineering new disulfide bond was improved (Fig. 1B, C), which the mutant A (F126C/S121C, Kabat numbering) increased by an average of around 20 % comparing that of wide type (WT). Although only one adjacent position of cysteine was different in the CH1 or CL fragment of h8B-BsAb-2 A, B and C, the result showed obvious incomplete antibodies in expression of mutant B and C (Fig. 1B). The cysteines mutation of D-G displayed with favorable formation of monomeric BsAbs, however, expression level of the mutant F and G significantly declined as shown in Fig. 1D.

Then we measured the thermal stability of BsAbs by differential scanning calorimetry (DSC). Values of a midpoint temperature of thermal unfolding transition (Tm) of mutant A, B and E had a slight increase, and other mutants revealed no benefit to thermostability compared with WT (Table 1). SK-GPC3 cells (Glypican-3) and PBMCs (CD3 positive) were used to determine the binding affinity of BsAbs by flow cytometry. All BsAbs showed similar affinities (Table 1), which suggested that the introduced disulfide bond had no effects on antibody-antigen interaction. Besides, the construction of the IgG-scFv BsAb remained a similar affinity to two antigens of parent monoclonal antibodies (data not shown). Therefore, residues P126 (CH1) and S121 (CL) were replaced to cysteine that could be the best position to form interchain disulfide bridge for a tetravalent IgG-scFv BsAb here.

**Table 1:** Thermostability and affinity of BsAbs with single disulfide bond introduction. The T_m_ values of BsAbs were obtained from a single determination, and the values of affinity were shown as mean ± SD (*n* = 3)

Antibody (h8B-BsAb-2)	WT	A	B	C	D	E	F	G
Thermostability (T_m_, ℃)	67.6	68.7	68.4	67.8	67.8	68.2	67.3	67.8
Affinity to Glypican-3 (nM)	1. 9 ± 0.1	2.4 ± 0.1	3.2 ± 0.3	2.2 ± 0.1	2.6 ± 0.2	4.5 ± 0.4	6.6 ± 0.2	2.8 ± 0.1
Affinity to CD3 (nM)	48.4 ± 6.2	42.1 ± 3.6	52.9 ± 5.4	85.3 ± 7.0	68.9 ± 3.2	170.2 ± 23	88.8 ± 6.5	94.0 ± 9.2

### Disulfide bonds construction in Fab domain of tetravalent T-cell engaging BsAbs

**Fig. 2:**
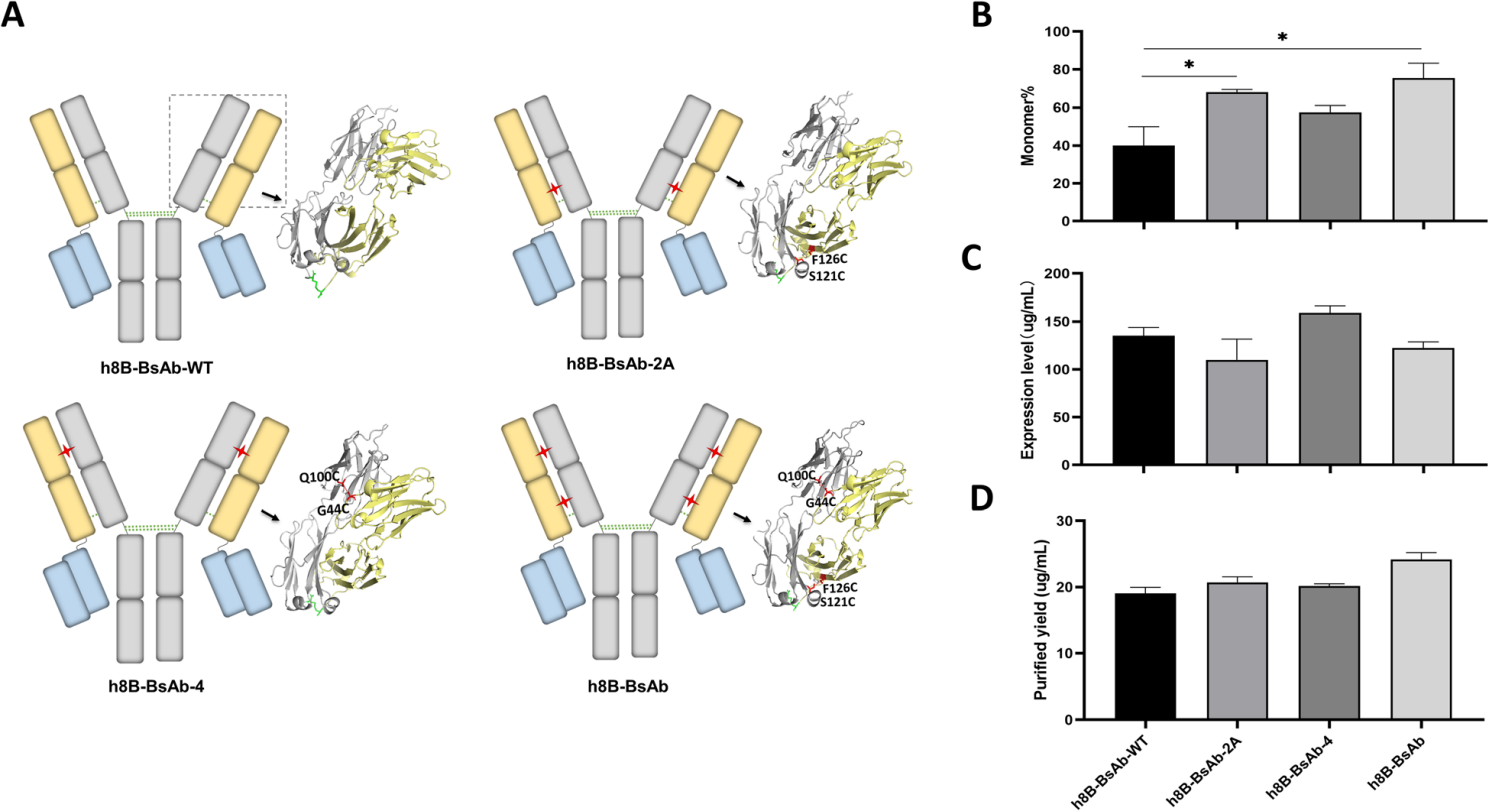
Single or double disulfide bonds introduction in the Fab domain of tetravalent BsAbs against glypican-3 and CD3. (**A**) Schematic of four BsAbs with introduced zero, one or two interdomain disulfide bonds in Fab region. The residues in CH1_126_/CL_121_ and VH_44_/VL_100_ colored by red were mutated to cysteines. (**B**, **C**) Expression and (**D**) purified yield levels of four BsAbs. Mean ± SD (*n* = 3), *P < 0.05

To further improve the stability and promote more monomeric BsAb formation, we introduced a disulfide bond (VH_44_-VL_100_) between variable domains of Fab, which several engineered molecules have proved to stabilize VH/VL interface [12, 20–22]. Thus, T-cell engaging BsAbs with IgG-scFv format were engineered, where one or two additional disulfide bridges were built in the Fab domain by substitution of VH_44_-VL_100_ or/and CH1_126_-CL_121_ residues with cysteines (Fig. 2 A). Results showed that h8B-BsAb with two engineered disulfide bonds could drive more formation of monomeric BsAbs during expression, though no significant increase in comparison of single disulfide bond introduction of h8B-BsAb-2 A and h8B-BsAb-4 (Fig. 2B). In addition, disulfide-introduction may affect the level of expression of BsAbs, but the purified yield had relatively improved by reducing unpaired chains (Fig. 2 C, D).

**Fig. 3:**
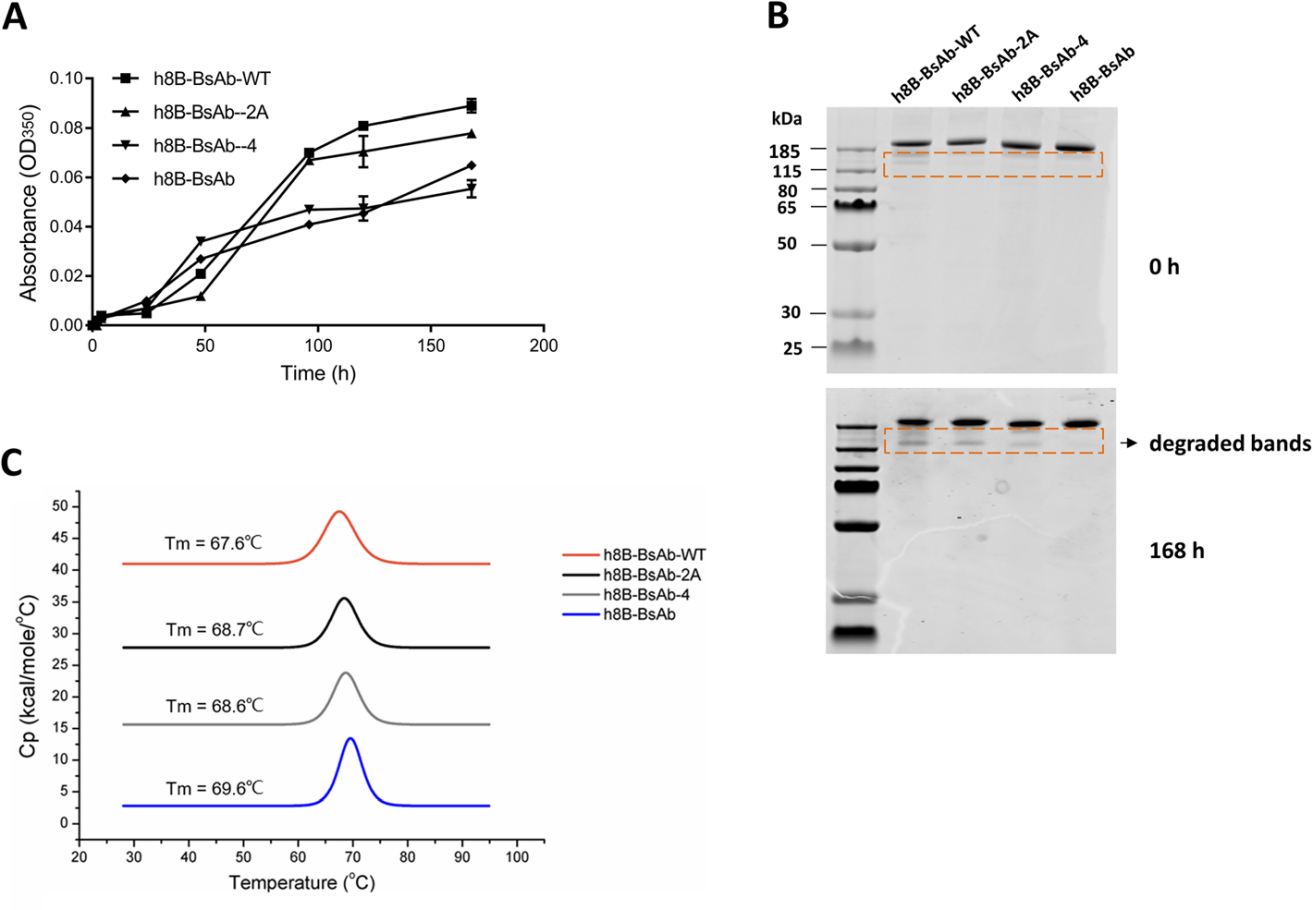
The stabilities of h8B-BsAb-WT, h8B-BsAb-2 A, h8B-BsAb-4 and h8B-BsAb. (**A**) the aggregation tendency of BsAbs was tested by turbidity assay. Antibodies were vortexed for up to 168 h at 25ºC and recording the absorbance (595nm) at various time points. (**B**) Antibodies without (0 h) or with a vortex (168 h) were analyzed by non-reducing SDS-PAGE. The red dotted line indicated the degradation of BsAbs. (**C**) The thermostability of BsAbs was measured by differential scanning calorimetry

Next, we compared the stabilities against aggregation and degradation of the tetravalent BsAbs under mechanical stresses. The purified BsAbs were evaluated by turbidity assay under 1400 rpm shaking for up to 168 h at 25 °C, and the increase rate of the absorbance at 350nm in h8B-BsAb-WT and BsAb-2 A was faster than other two BsAbs (Fig. 3 A). Thus, the h8B-BsAb and h8B-BsAb-4 comparatively displayed greater aggregation-resistance under these conditions. SDS-PAGE analyzed the antibodies after 168 h agitation, and no degradation was observed in h8B-BsAb while the degraded protein fragments distinctly found in h8B-BsAb-WT, h8B-BsAb-2 A and h8B-BsAb-4 (Fig. 3B). The DSC profile of the purified BsAbs (Fig. 3 C) exhibited that h8B-BsAb had higher thermal stability with a Tm value of 69.6 °C similar to that of parent anti-Glypican-3 antibody. The h8B-BsAb-2 A and h8B-BsAb with single disulfide bridge introduction had almost the same midpoint melting temperature. We are surprised that BsAbs with one or two engineered disulfide bonds getting small increases of thermal stability, which may be due to either no planned disulfide or to only a small percentage of disulfide bonds actually forming. So, we performed 5,5’-Dithiobis (2-nitrobenzoic acid) (DTNB) assay and fourier transform infrared (FITR) spectroscopy analysis to quantify the stoichiometry of free cysteines present to assist in confirmation of disulfide formation. There were less cysteines or thiol groups (–SH) detected as showed in DTNB and FTIR results (Supplementary Figs. 1, 2) with no significant differences between these four antibodies, which mean that most of cysteines formed disulfide bonds. In the comparison of h8B-BsAb-WT, we concluded that engineered two disulfide bonds in the Fab domain of tetravalent IgG-scFv could apparently improve stabilities against mechanical stresses, and had a slight improvement of thermo-resistance.

**Fig. 4:**
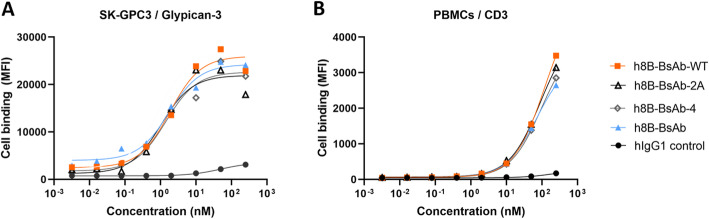
The affinity of the four BsAbs against glypican-3 and CD3. (**A**) The SK-GPC3 cells and (**B**) human peripheral blood lymphocyte (PBMCs) as target cells were incubated with BsAbs for flow cytometry analysis. MFI: mean fluorescence intensity

**Fig. 5:**
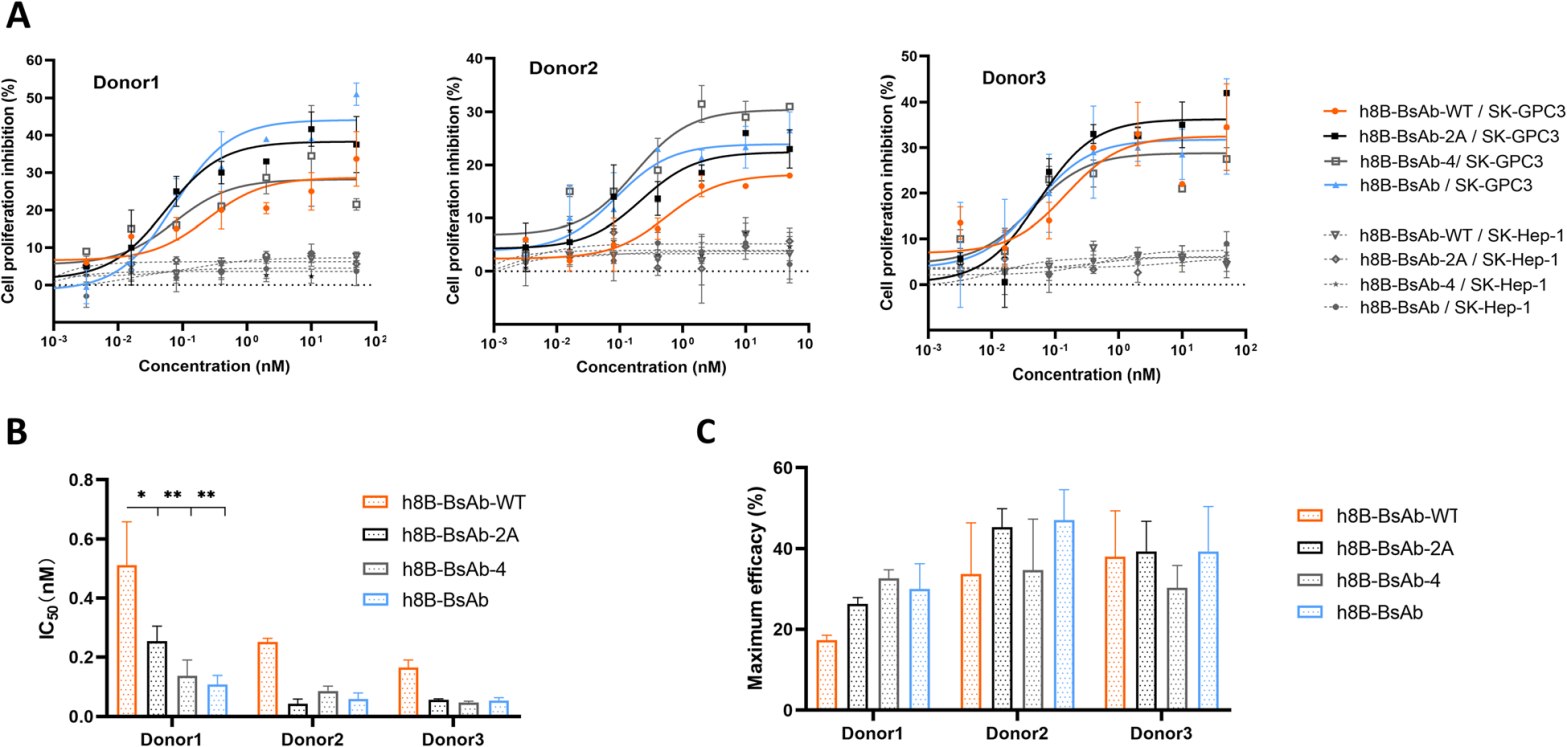
Inhibition of cell growth by the BsAbs targeting glypican − 3 and CD3. The four BsAbs were cultured with SK-GPC3 cells or SK-Hep-1 cells (target cells, T) and PBMCs (effector cells, E) at a E:T ratio of 10:1 for 48 h. (**A**) Inhibition of proliferation was measured by CCK-8 assay. Data analysis of IC50 (**B**) and Maximum efficacy (**C**) performed by Prism Version 8.0. Mean ± SD (*n* = 3), *P < 0.05, **P < 0.01. SK-GPC3 cells: glypican-3 positive; SK-Hep-1 cells: glypican-3 negative

To confirm the biological activity of BsAbs, cell binding assay was first performed by flow cytometry using glypican-3 and CD3 positive cells to incubate with diluted antibodies. Results demonstrated that one or two disulfide bonds introducing in a BsAb had no effect upon the cell binding affinity (Fig. 4 A, B), and even had a slight increase compared with h8b-BsAb-WT (Table 2). Then, we evaluated the T- cell redirection activity of the four BsAbs that can bridge with their target cells and activate T cells for cell killing [31]. Target cells and effector cells (PBMCs) were mixed at a ratio of 1:10 for incubation with BsAbs. The cytotoxicity of BsAbs to target cells was measured as growth inhibition resulting from reduced proliferation over the course of 48 h. Results shown in Fig. 5 A indicated that the four BsAb induced an antibody concentration-dependent cell growth inhibition in SK-GPC3 cells (GPC3 positive) but no apparent inhibition of SK-Hep-1 (GPC3 negative). By comparing potency (concentration of half-maximum inhibition, IC_50_ value) and maximum inhibitory effect, h8B-BsAb had preferable inhibitory effects at saturating concentration than that of h8B-BsAb-WT, albeit only minor differences in cytotoxicity of h8B-BsAb-2 A, h8B-BsAb-4 and h8B-BsAb (Fig. 5B, C).

**Table 2:** Binding affinity of BsAbs to antigens. Mean ± SD (*n* = 3)

Antibody	h8B-BsAb-WT	h8B-BsAb-2 A	h8B-BsAb-4	h8B-BsAb
Glypican-3 (nM)	1. 9 ± 0.1	2.4 ± 0.1	3.2 ± 0.3	2.2 ± 0.1
CD3 (nM)	48.4 ± 6.2	42.1 ± 3.6	52.9 ± 5.4	85.3 ± 7.0

### Engineering double disulfide bonds in different tetravalent T-cell engaging BsAbs

**Fig. 6:**
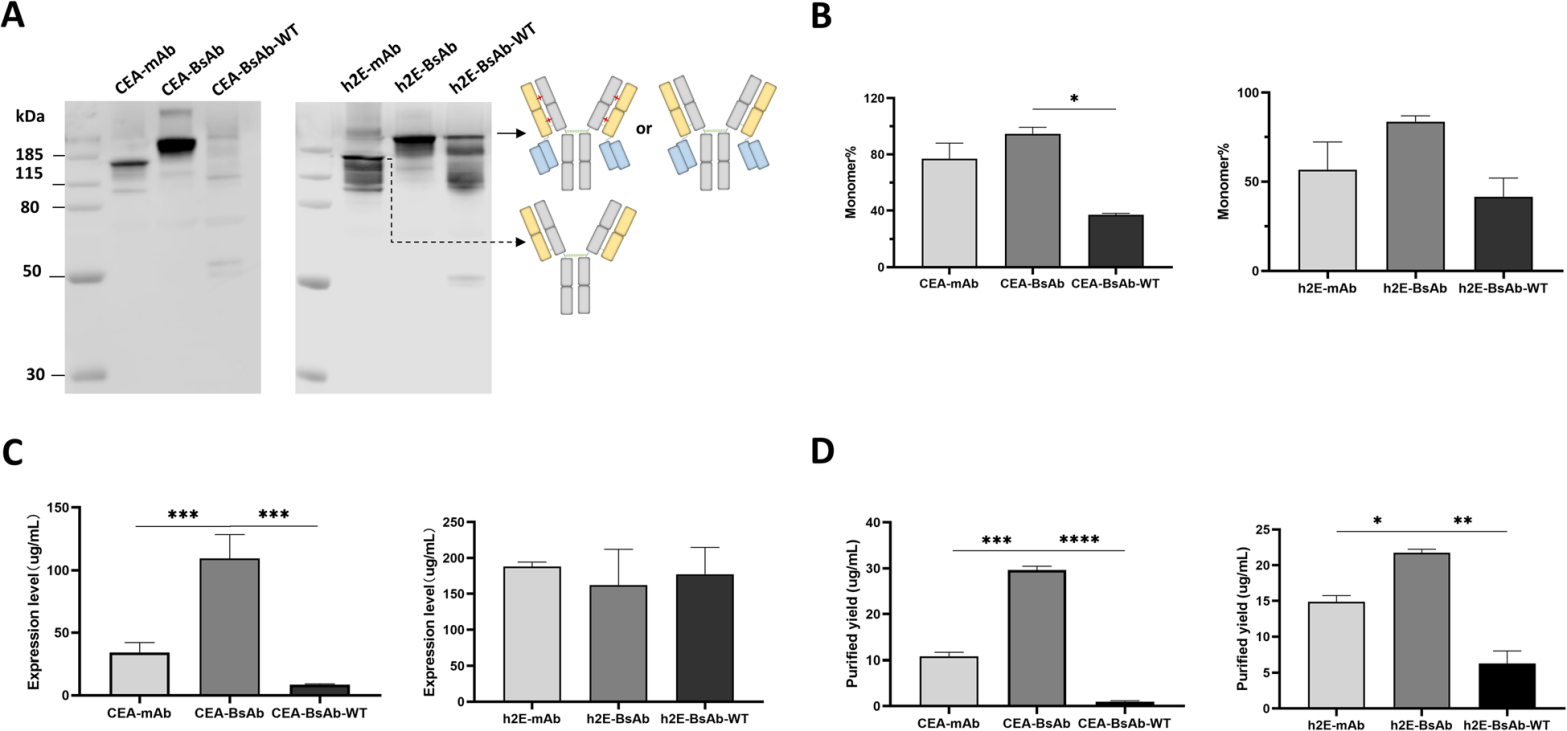
Interchain disulfide bonds introduced in different tetravalent T-cell engaging bispecific antibodies. (**A**) Expression of monoclonal antibodies (CEA-mAb and h2E-mAb) and BsAbs (CEA-BsAb-WT, CEA-BsAb, h2E-BsAb-WT and h2E-BsAb), was displayed by WB under non-reducing condition. Quantitative analysis for levels of monomer (**B**), expression (**C**) and purified yield (**D**). Mean ± SD (n = 3), *P < 0.05, **P < 0.01, ***P < 0.001, ****P < 0.0001

We further investigated whether the cysteine mutation of VH_44_-VL_100_ and CH1_126_-CL_121_ can apply to other T-cell engaging IgG-scFv molecules with different antigen-binding sites to favor the formation of a monomeric BsAb. Two humanized IgG1 monoclonal antibodies (CEA-mAb [32] and h2E-mAb generated from our laboratory) targeting carcinoembryonic antigen (CEA) and glypican-3, respectively, were used to construct the bispecific IgG-scFv antibodies: the CEA-BsAb-WT and h2E-BsAb-WT. Then, the CEA-BsAb and h2E-BsAb were built by introducing two interchain disulfide bonds in Fab domains of CEA-BsAb-WT and h2E-BsAb-WT. All six antibodies were independently expressed three times, and the representative result showed that CEA-BsAb and h2E-BsAb had prominent formation of monomers (Fig. 6 A, B). In contrast, few CEA-BsAb-WT molecules were detected (Fig. 6 A). The light chain of CEA-mAb fused a scFv fragment might affect the formation of the native interdomain disulfide bridges and result in instability of single chain for existence during cell culture. The level of expression and purified yield of CEA-BsAb was notably higher than that of CEA-mAb and CEA-BsAb-WT (Fig. 6 C, D). Although the expression of h2E-mAb, h2E-BsAb-WT and h2E-BsAb had no significant difference, h2E-BsAb was purified with high yield (Fig. 6 C, D). We measured the binding activity of these antibodies, and results confirmed that the BsAbs with engineered double disulfide bonds can maintain the original affinity to antigens (Table 3 A, B), similarly behaved like h8B-BsAb as described.

**Table 3:** Binding affinity of BsAbs. Mean ± SD (*n* = 3)

a. Affinity of CEA-mAb and BsAbs to antigens. CEA: carcinoembryonic antigen.		
Antibody	Affinity (nM)	
	Binding to CEA	Binding to CD3
CEA -mAb	4.7±0.3	-
CEA -BsAb	2.3±0.4	50.7±7.4
CEA -BsAb-WT	4.6±0.1	97.1±5.2
b. Affinity of h2E-mAb and BsAbs to antigens.		
Antibody	Affinity (nM)	
	Binding to Glypican-3	Binding to CD3
h2E-mAb	47.0±4.1	-
h2E-BsAb	80.5±7.6	49.3±2.3
h2E-BsAb-WT	64.2±5.5	25.1±9.7

## Discussion

The T-cell engaging bispecific antibody with 30 years of development has been demonstrated as a potential strategy for cancer therapy. Also, the BsAb format appeared to effect BsAb potency significantly. A prior study showed that tumor cell killing of scFv-based BsAb design had over ten-fold better than that of Fab-based BsAbs [8]. T-cell engaging IgG-scFv BsAbs with dual bivalency had superior in vitro and vivo cytotoxicity in contrast to ordinary bivalent heterodimer [10, 11, 14]. Additionally, the T cell binding domain (scFv) connected to the C terminal of the anti-tumor IgG light chain exhibited more potent T cell cytotoxicity, and these two functional domains were placed on the same side [14]. However, this configuration of light chain fused a scFv might influence the native stability of IgG by changing bonding distance of interdomain disulfide bonds. It has been reported that an 18 amino acid peptide or scFv linked to the CL domain of IgG more tended to dissociation to under heat-stress [15]. Our early results also found plenty of unpaired light and heavy chains during the expression of BsAb, which easily leads to low production. Therefore, the problems as described may limit further clinical application.

Disulfide-stabilization strategy has been proved to rise protein stabilities including IgG molecules with inter or intra-domain disulfide bonds introducing [17, 22, 33, 34]. In this study, we engineered a series of mutations in the CH1/CL domain of BsAbs to add a disulfide bond to facilitate more monomers formation. The position of F126 (CH1) and S121(CL) by replacement of cysteines exhibited the highest thermostability and proportion of monomers in cellular supernatant, where unexpectedly are consistent with a recent report describing alteration of the disulfide bridge in Fab domain to reduce mispairing of the heavy and light chain of a monovalent bispecific antibody [26]. In a previous study, site-direct mutagenesis at V177C and Q160C of an adalimumab Fab was performed to increase the thermal stability [16], while our similar mutational position of V173C/Q160C showed low thermostability and expression level. It’s reported that engineering a disulfide bond in fragment with simple structure (e.g., scFv, Fab) often observed more remarkable improvement in terms of thermal stability [12], but we didn’t see the same introduction had a significant increase in full-length antibodies. Although there was one adjacent position different of mutant A (F126C/S121C) with mutant B (F126C/Q124C) and C (P127C/S121C), as well as the carbon distance of disulfide bond was within the estimated range (C_α_-C_α_≤7.0 Å, C_β_-C_β_≤4.7 Å), we think this results showed apparent differences in the level of monomers (Fig. 1B) which attribute to incomplete disulfide bonds. It demonstrated that F126 and S121 are optimal positions to engineer an interchain disulfide bridge in CH1/CL domain of IgG.

Introducing two additional disulfide bonds in the Fab domain enhanced more light and heavy chains assembly for monomeric BsAbs, especially in the case of the CEA-BsAb with dramatically increase. Disulfide bonding is an important post-translational modification in proteins, including antibodies and extra disulfide bonds engineered may decrease yield due to the formation incorrect disulfide bonds during the folding process [35]. However, it seems that no noticeable reduction in the position of VH_44_-VL_100_ and CH1_126_-CL_121_ by adding new interdomain disulfide bonds, and all BsAbs (h8B-BsAb, CEA-BsAb and h2E-BsAb) engineered double disulfide bonds exhibited higher production than WT format. Strangely, the expression level of CEA-BsAb-WT was extremely low (Fig. 6) despite no engineered disulfide bonds in its Fab domain. Then we expressed only single or two complete chains by HEK-293 cells and found that few light chains and heavy chains with cystine mutation detected in cell supernatant in CEA-BsAb and CEA-BsAb-WT, but complete CEA-BsAb molecules were still distinctly observed (data not shown). As the interchain disulfide bonds are not efficiently formed, the single chains with free sulfhydryls exposure to solvent are more prone to degradation [36]. Although all described antibodies had no impact or reduce a binding activity, BsAbs without engineered disulfide bonds of the Fab domain tended to aggregation and degradation under mechanical stresses. In light of these results, the generalizability for other sequences of antibodies, by substitution of VH_44_-VL_100_ and CH1_126_-CL_121_ residues with cysteines, were roughly validated. There is a considerable variation in the inhibitory effect levels among different donors due to the individual difference. Nonetheless, the h8B-BsAb had higher cytotoxicity to target cells than h8B-BsAb-WT in general (Fig. 5).

In summary, we designed several tetravalent T-cell engaging bispecific antibodies with IgG- scFv format targeting different antigens. By two additional interchain disulfide bonds introduction in the Fab region, BsAbs had provided better biophysical and biological activities over wide types.

## Supplementary Information

**Additional file 1.** 

## Data Availability

The datasets supporting the conclusion of this article are included within the article and the additional files.

## Abbreviations

BsAb: Bispecific antibody
scFvs: Single-chain variable fragments
IgG: Immunoglobulin G
Fab: Antigen binding fragments
VH: Heavy chain
VL: The light chain
DSC: Differential scanning calorimetry
FCM: Flow cytometry
BLI: Biolayer interferometry
WT: Wide type
Tm: Temperature of thermal unfolding transition
5,5’- DTNB: Dithiobis (2-nitrobenzoic acid)
FITR: Fourier transform infrared
PBMCs: Peripheral blood lymphocyte
GPC3: Glypican-3
CEA: Carcinoembryonic antigen
