# Supplementary material for: Structural design of tetravalent T-cell engaging bispecific antibodies: improve developability by engineering disulfide bonds
Source: J Biol Eng. 2021 Jun 29;15:18. doi: 10.1186/s13036-021-00272-7 (PMC8243740; doi:10.1186/s13036-021-00272-7)
Supplement: Supplementary file 1 — Additional file 1. [file 13036_2021_272_MOESM1_ESM.docx]

**Supplementary materials**

**Supplementary methods**

**DTNB titration of antibodies**

To investigate whether the engineered disulfide bonds are actually formed, we use 5,5'-Dithiobis (2-nitrobenzoic acid) (DTNB) titration of (unfolded) protein to quantify the stoichiometry of free cysteines or thiol groups (–SH) present. The DTNB reagent was purchased in ALADDIN (Shanghai, China) and was adjusted to 1mg/mL by 100mM PBS buffer (pH 7.2-7.4). 100uL antibodies with 2mg/mL or 100uL Dithiothreitol (DTT) with 0.015mg/ml are mixed with 10uL DTNB for 3min at room temperature. Then absorption value of mixtures was measured by a visible-ultraviolet spectrophotometer (BioTek) at 412nm.

**Fourier transform-infrared spectrometer (FTIR)**

Antibodies were desalted by dialyzing with water and then treated with vacuum freeze-drying to be solid powder. The antibodies films were tested using Fourier infrared transform spectroscopy of Nicolet iS10 FTIR (Thermo Nicolet Corporation). The scan range was from 4000 to 400 cm^−1^ and 1000 to 400 cm^-1^ [and](javascript:;) this test was performed three times independently.

**Supplementary Figures**


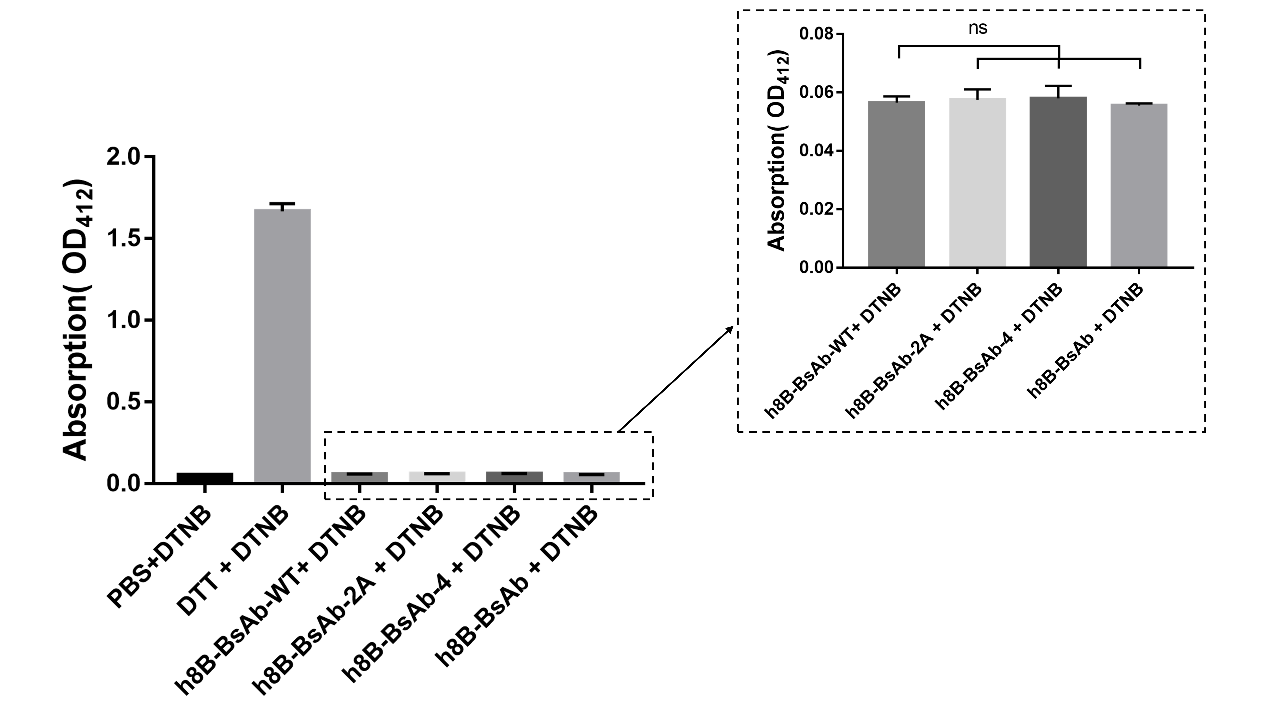


**Supplementary Fig.1** DTNB titration of unfolded antibodies. Group of PBS+DTNB and DTT+DTNB were as negative and positive control, respectively.


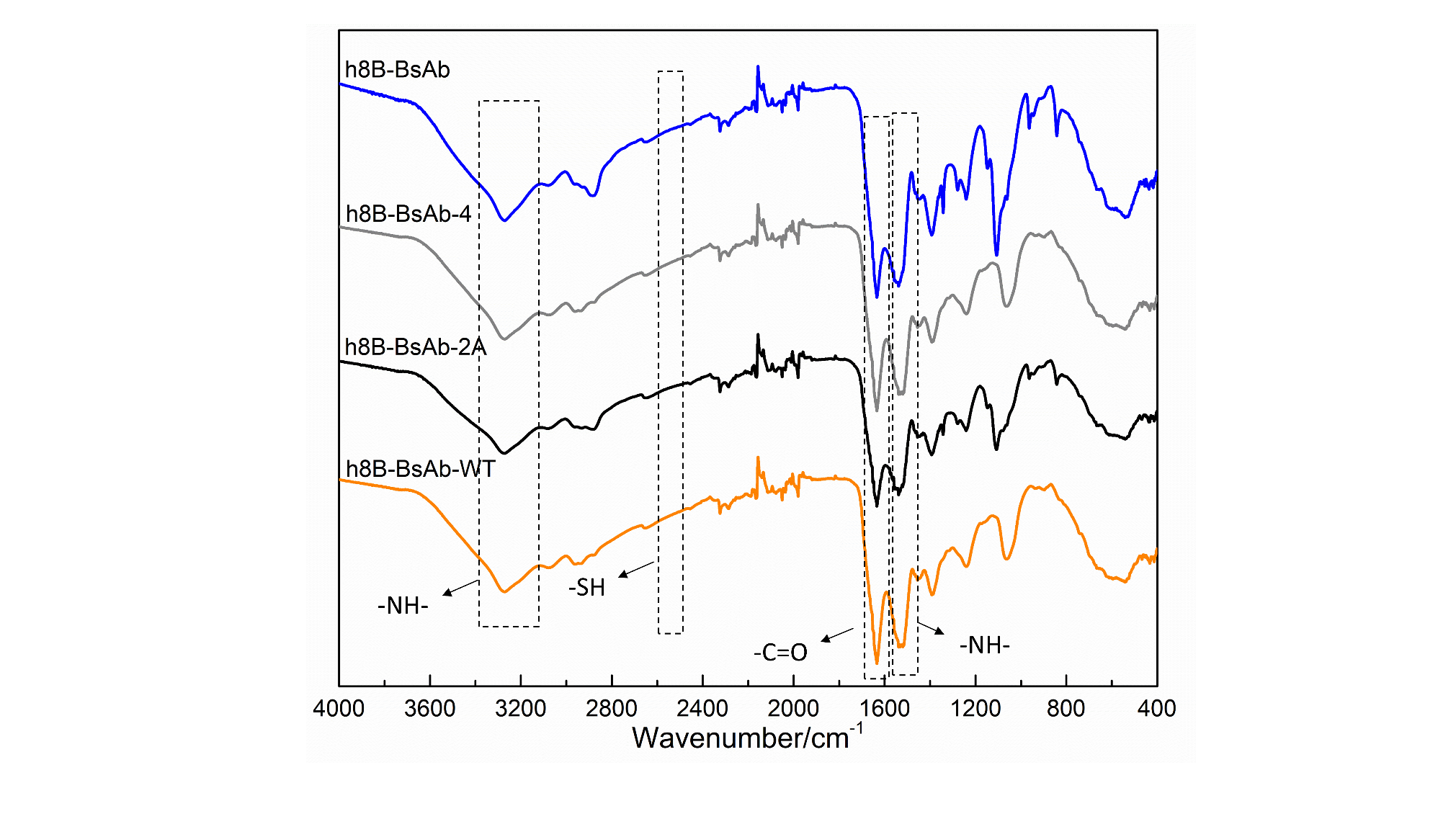


**Supplementary Fig.2** FTIR of unfolded antibodies. No characteristic peaks of free thiol groups (-SH, 2500-2600 cm^-1^) observed in FITR.
